# Characterization of Bioactive Compounds Having Antioxidant and Anti-Inflammatory Effects of Liliaceae Family Flower Petal Extracts

**DOI:** 10.3390/jfb13040284

**Published:** 2022-12-08

**Authors:** Neha Kaushik, June-Hyun Kim, Linh Nhat Nguyen, Nagendra Kumar Kaushik, Kyung-A Choi

**Affiliations:** 1 Department of Biotechnology, The University of Suwon, Hwaseong 18323, Republic of Korea; 2 Laboratory of Plasma Technology, Institute of Materials Science, Vietnam Academy of Science and Technology, 18 Hoang Quoc Viet, Hanoi 100000, Vietnam; 3 Plasma Bioscience Research Center, Applied Plasma Medicine Center, Department of Electrical and Biological Physics, Kwangwoon University, Seoul 01897, Republic of Korea; 4 National Institute of Medical Welfare, Kangnam University, Yongin 16979, Republic of Korea

**Keywords:** lily bulbs, flower petal extracts, macrophage cell line, anti-inflammatory, antioxidant

## Abstract

Beneficial natural products utilized in cosmetics formulation and pharmaceutical applications are of enormous interest. Lily (Lilium) serves as an essential edible and medicinal plant species with wide classification. Here, we have performed the screening of various extracts that were prepared from flower petals grown from the bulbs of eight Lilium varieties, with a viewpoint to their applicability as a viable source of natural anti-inflammatory and antioxidants agent. Interestingly, our findings indicated that all ethanol and water extracts exhibited a substantially differential spectrum of antioxidant as well as anti-inflammatory properties. Specifically, Serrano showed a close similarity among ethanol and water extracts among all tested lily petal extracts. Therefore, to obtain a detailed analysis of chemical compounds, liquid chromatography–mass spectroscopy was performed in ethanolic and water extracts of Serrano petals. Together, our preliminary results indicated that lily petals extracts used in this study could serve as a basis to develop a potential new whitening agent with powerful antioxidant and anti-inflammatory properties for medicinal, functional food, and cosmetic applications.

## 1. Introduction

Recently, plant extracts have been attracting huge attention in scientific society, being considered active constituents of cosmetic formulations for their health and beauty advantages [1]. Consumption of natural cosmetic products is progressively becoming a reality in the cosmetics industries since customers believe those to be “environmentally friendly harmless products” characterized by the lack of synthetic components. Health and beauty-related plant-derived extracts are used for cosmetic purposes owing to their antibacterial, antioxidant, antiaging, UV protection, and anti-inflammatory activities [1]. Lily is a widely cultivated plant that belongs to the Liliaceae family and has immense medicinal, ornamental, as well as edible value [2,3,4]. The genus Lilium, which consists of more than 90 species/varieties, is native to Asian, American, and European countries. It is generally propagated through bulbs; however, few species can also be grown via seeds. Many phytochemical findings have revealed that in addition to the essential metabolites, the chemical components of Lilium plants also comprise several secondary metabolites, containing phenols, flavonoids, sterols, alkaloids, and saponins [5,6,7,8], which are essential sources of medicinal complexes that might be applied to treat different diseases, including tumors and hyperglycemia [9,10,11]. Lily bulbs have a long, well-known history of medicinal usages, including for sedative effects [12], ultraviolet (UV) absorption, cancer suppression, and free radical scavenging, and in skin care, for anti-wrinkle effect [1,13,14] as well as anti-inflammatory properties [15,16,17]. Inflammation is an instant reaction of organisms upon injuries or in the presence of outside foreign substances, i.e., pathogens. Several studies have suggested that macrophages play an essential role in the host’s defense against harmful bodies [18,19]. During this process, macrophages are the main immune cells originating from monocytes and placed throughout the body tissues [20]. These cells actively participate in an inflammatory response and assist in antigen presentation, immune modulation, phagocytosis, and tissue repair [21]. Interestingly, the anti-inflammatory effect of methanolic extracts of Lilium lancifolium was also observed in stimulated murine macrophages [17]. Similarly, Luo et al. also mentioned the role of methanol lily bulb extracts in anti-inflammatory and antibacterial responses [22]. Even though more than 25 species of *Lilium* have been applied as food and traditional medicine globally, the investigation of the active constituents of *Lilium* is concentrated on only a limited species or varieties, such as *Lilium davidii* var. willmottiae, *Lilium brownie*, *Lilium lancifolium*, *Lilium pumilum*, and *Lilium candidum*. [10,23,24]. Notwithstanding a long history of usage, there are few valuable reports on the active constituents of diverse wild lily species; however, there are no details on comparative studies among different varieties. This is not beneficial to the medicinal application of these valued species. Reasonably, more studies exploring such activities in Lilium species are needed.

Tyrosinase, a glycoprotein enzyme [25] that catalyzes aerobic tyrosine oxidation to form melanin pigment, is broadly dispersed in nature and has been observed in plants [26,27]. It is a key enzyme involved in the several essential initial rate-determining steps during the melanin biosynthesis pathway inside humans. To date, several whitening constituents have a mode of action for tyrosinase enzyme inhibition. In this regard, we estimated the degree of tyrosinase enzyme inhibition in vitro using established methods mentioned earlier [28]. Currently, the utilization of tyrosinase inhibitors is becoming gradually popular in the cosmetic industries, probably owing to their skin-whitening effects, notwithstanding the wide-ranging studies on skin-lightening agents and overcoming hyperpigmentation. The tyrosinase-inhibiting agents face restrictions because of less stability, high toxicity, and reduced skin penetration [29]. Nature-inspired tyrosinase inhibitors are commonly considered free of detrimental side effects and may likely be formed at lower costs, particularly when rich sources are recognized [30]. Based on the above literature, the present study has been carried out to investigate the antioxidant and anti-inflammatory potential of eight different lily varieties. Prominent compounds were explored using liquid chromatography–mass spectroscopy in potential lily extract candidates for further characterization. Positively, this report would offer the necessary experimental indication of the anti-inflammatory and antioxidant potential of lily flower extracts for future development and consumption of these lily varieties in cosmetics as well as medicinal applications.

## 2. Materials and Methods

### 2.1. Reagents/Chemicals

All reagents or chemicals for anti-tyrosinase and antioxidant assays were purchased from Sigma-Aldrich (Seoul, Korea) or Duksan Chemicals (Ansan, Korea), and used without additional purification in experiments.

### 2.2. Collection of Flowers and Extract Preparation

Several lilies grown from bulbs were planted at the Research Institute of Woori Flower, Seed & Seeding Co., Ltd., Gwacheon, Korea (37.4292° N, 126.9874° E). All eight lily varieties used in this study are as follows: Hybrid Orienpet Lily ‘Yelloween’, *Lilium longiflorum* ‘Woori Tower’, *Lilium longiflorum* ‘Show up’, *Lilium longiflorum* ‘Cali’, Daylily Hemerocallis ‘Pink Playmate’, Oriental Hybrid Lily ‘Pinnacle’, Hybrid Lily ‘Faconnable’, and Orienpet Lily ‘Serrano’. Most of these species originate from the Netherlands. For biological studies, flower petals were used from these lily flowers and extracted with 70% ethanol or distilled water (DW) as previously described [31]. Briefly, all Lily flowers originating from the Netherlands were obtained from Woori flower, Seed and Seedling, Co., Ltd., Korea. To prepare the extracts, flower petals were collected and dried in the oven (Jisico, Seoul, Korea). Afterward, the samples were homogenized with a mixer (Hanil, Bucheon, Korea), extracted with 70% ethanol (Duksan Chemicals, Ansan, Korea) or distilled water, concentrated with a rotary evaporator (Eyela, New York, NY, USA), and lyophilized with a freeze dryer (Ilshin Bio, Dongducheon, Korea).

### 2.3. Cell Culture and Extract Treatment

Raw 264.7 macrophage cell lines (KCLB, 40071) were cultured in Dulbecco’s Modified Eagle’s Medium (DMEM) supplemented with 1% penicillin/streptomycin and 10% fetal bovine serum. Cultured cells were maintained in a humidified atmosphere using 5% CO_2_ at 37 °C. After reaching the required confluency, cells were seeded for experiments and exposed to different concentrations of flower extracts. Stocks and their dilution were made in DW for desired treatments.

### 2.4. Antioxidant Assays

The effects of the lily flower extract on 1,1-diphenyl-2-picrylhydrazyl (DPPH) radicals were assessed according to the procedure mentioned in the reported literature [32]. Briefly, 1 mL sample solution was mixed with the 4 mL of a 0.004% DPPH dissolved in methanol solution. Ascorbic acid was included as a positive control to quantify DPPH activity in extract samples. After 30 min incubation in dark conditions, the color developed in test samples was measured at 517 nm absorbance using an ELISA reader (Epoch, Biotek Instruments, Inc., Santa Clara, CA, USA).

### 2.5. Cell Viability Assay

MTT (Sigma-Aldrich, Korea) assay was employed to measure cell viability in macrophage cells. To this end, RAW264.7 cells were seeded at a density (1 × 10^4^ cells/well) and stimulated with lipopolysaccharide (LPS-40 µg/mL) for 2 h before extracting samples. Red charm has been used as a positive control. After desired time (48 h), 20 µL MTT stock (5 mg/mL) was added to all wells, followed by incubation for an additional 4 h. Absorbance was detected at 570 nm to calculate the optical density that was directly proportional to the number of viable cells in test samples [33].

### 2.6. Nitric Oxide Assay

The quantity of nitric oxide (NO) produced was estimated from the aggregation of the nitrite using the Griess reagent assay. In this test, the 100 µL cell culture supernatant was collected from treated and untreated samples and combined with 100 µL Griess reagent, and the absorbance was determined at 540 nm [34].

### 2.7. In Vitro Tyrosinase Inhibition Assay

The melanin synthesis pathway in melanocytes suggests that tyrosine is hydroxylated to 3,4-dihydroxyphenylalanine (DOPA) by the enzyme tyrosinase, and melanin is synthesized through the process of dopaquinone and dopachrome. It is a method of measuring the whitening effect by measuring tyrosinase activity. Briefly, after dissolving the sample in ethanol, dilute it further to a suitable concentration range to reduce tyrosinase activity. After the addition of 105 μL of 0.1 M phosphate buffer (pH 6.5), 20 μL of test extracts solution, and 20 μL (2000 U/mL) of mushroom tyrosinase solution into the Eppendorf tube, add 5 µL of 0.2 mM tyrosine solution into this solution and incubate for 15 min at 37 °C. Immediately, absorbance was analyzed at 490 nm by an ELISA reader as previously described [32]. For the blank sample, 0.1 M phosphate buffer (pH 6.5) was mixed instead of the test sample solution. The IC_50_ concentration of each extracted sample is calculated by an appropriate program when the activity inhibition rate is 50%. Additionally, arbutin was used as a positive control (data not shown).

$$\mathrm{Tyrosinase}\;\mathrm{activity}\;\mathrm{inhibition}\;\mathrm{rate}\;\left(\%\right)=100-[\frac{\left(b-b’\right)}{\left(a-a’\right)}\times 100]$$

where a is the absorbance after the reaction of blank sample solution, b is the absorbance after the reaction of the sample solution, and a’, b is the absorbance measured by substituting buffer for tyrosinase. For the detection of tyrosinase inhibitory characteristics of the petal ethanolic and DW extracts of various lily species flower extracts, a mushroom tyrosinase inhibition test was performed with tyrosine solution at a different concentration ranging from 500 to 5000 μg/mL for 15 min at 37 °C, and the absorbance was measured.

### 2.8. Liquid Chromatography Mass Spectroscopy

The LC-MS analysis of lily extracts was carried out using the Ultimate 3000 RSLC System/Q-EXACTIVE ORBITRAP PLUS MS (Thermo Fisher, San Jose, CA, USA). The analysis was performed by positive-mode electron spray ionization (ESI). The mass range was selected from 67 to 1000 m/z with an isolation window of 2 m/z. The capillary temperature was fixed at 320 °C. The collision-induced dissociation energy (CID) was set at 30 eV with a dynamic exclusion of 5 s. The spray voltage was set at 3.5 kV. The chemical structure, molecular formula, and molecular weight for each compound were confirmed using the open database from www.chemspider.com.

### 2.9. Statistical Analysis

Experimental data are expressed as the means ± SD of triplicates. Statistical comparison was performed either using two-way ANOVA with Dunnett corrections for multiple comparisons to untreated control as per experimental conditions using PRISM9 software (GraphPad Software, San Diego, CA, USA). Results were considered significant when * *p* < 0.05; ** *p* < 0.01; and *** *p* < 0.001.

## 3. Results

### 3.1. Antioxidant Activities of Liliaceae Family Flower Petal Extracts

**Figure 1:**
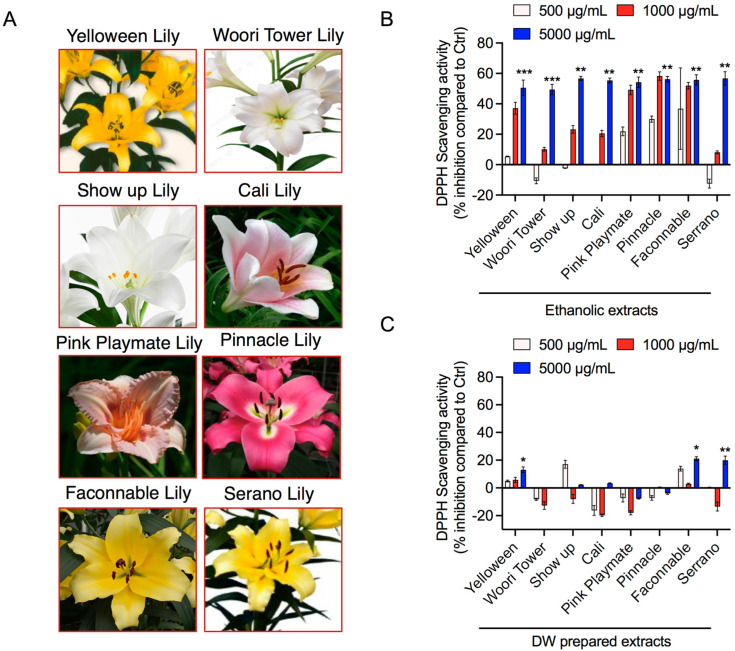
Effect of the lily flower petal extracts on the antioxidant capacity. (**A**) Photographs of different lily flowers used in this study. (**B**,**C**) DPPH assay of various lily flower petal extracts as per indicated panel prepared in ethanol and DW at 500, 1000, and 5000 µg/mL concentrations. Ascorbic acid (Sigma-Aldrich, Korea) was used as a control. Significance has been considered * *p* < 0.05, ** *p* < 0.05, *** *p* < 0.05.

In this study, we used eight different lilies, as shown in Figure 1A. Thaipong et al. demonstrated that all DPPH, ABTS, FRAP, and ORAC assays were well-correlated with ascorbic acid for antioxidant activity [35]. In our study, we have adopted the DPPH method to measure antioxidant activity in our samples among all these assays. Antioxidant effects on DPPH radical scavenging are usually due to their capacity for hydrogen-donating. In the current study, all lily bulb flower petal extracts were tested using various concentrations and were capable of quenching DPPH•. All tested lily flower extracts showed good antioxidant activity in a concentration-dependent manner. Especially, Yelloween, Pink playmate, Pinnacle, and Faconnable ethanol extracts showed a linear correlation with antioxidant activities in the 500, 1000, and 5000 µg/mL concentration range (Figure 1B). In the case of water extracts, pink playmate and Pinnacle did not show any antioxidant activities, whereas Yelloween and Faconnable showed significant antioxidant activities (Figure 1C). This shows that ethanol-soluble components (Figure 1B) from pink playmate and Pinnacle have much stronger antioxidant activities than their water-soluble components (Figure 1B). These results suggest that ethanol crude extracts from pink playmate and Pinnacle possibly include pro-oxidant constituents, which could participate with the antioxidants during reaction with DPPH radicals, but not with water extracts.

### 3.2. Tyrosinase Inhibition Properties of Liliaceae Family Flower Petal Extracts

**Figure 2:**
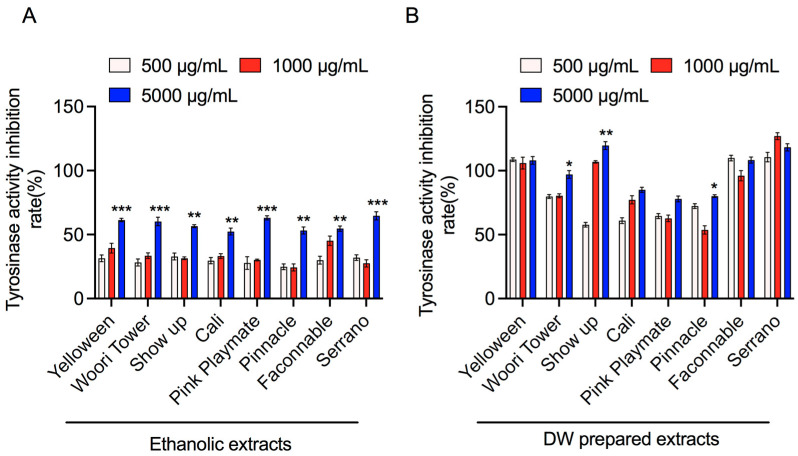
Effect of the lily flower petal extracts on the mushroom tyrosinase inhibitory activity. (**A**,**B**) Tyrosinase activity inhibition assay of various lily plant flower petal extracts as per indicated panel, prepared in ethanol and DW at 500, 1000, and 5000 µg/mL concentrations. Significance has been considered * *p* < 0.05, ** *p* < 0.05, *** *p* < 0.05.

For the detection of tyrosinase inhibitory characteristics of the ethanolic and DW extracts of various lily species flower extracts, a mushroom tyrosinase inhibition test was performed with tyrosine solution at a different concentration ranging from 500 μg/mL to 5000 μg/mL for 15 min at 37 °C, and the absorbance was measured as described above. Interestingly, all eight flower ethanol extracts (5000 μg/mL) showed 55–65% inhibition of tyrosinase activity in a dose-dependent fashion (Figure 2A), whereas water extracts showed 80–120% inhibition of tyrosinase activity in a dose-independent manner when 5000, 1000, and 500 μg/mL of flower extracts were used (Figure 2B), indicating that concentrations of water-soluble extracts have already reached the level that far exceeded the optimal concentration range to be able to measure tyrosinase inhibition assay. This result revealed that the identification of ethanol-soluble components in these flowers would give us a basis to develop a new powerful whitening agent.

### 3.3. Effect of Lily Flower Petal Extracts on the Viability of LPS-Stimulated Macrophages

**Figure 3:**
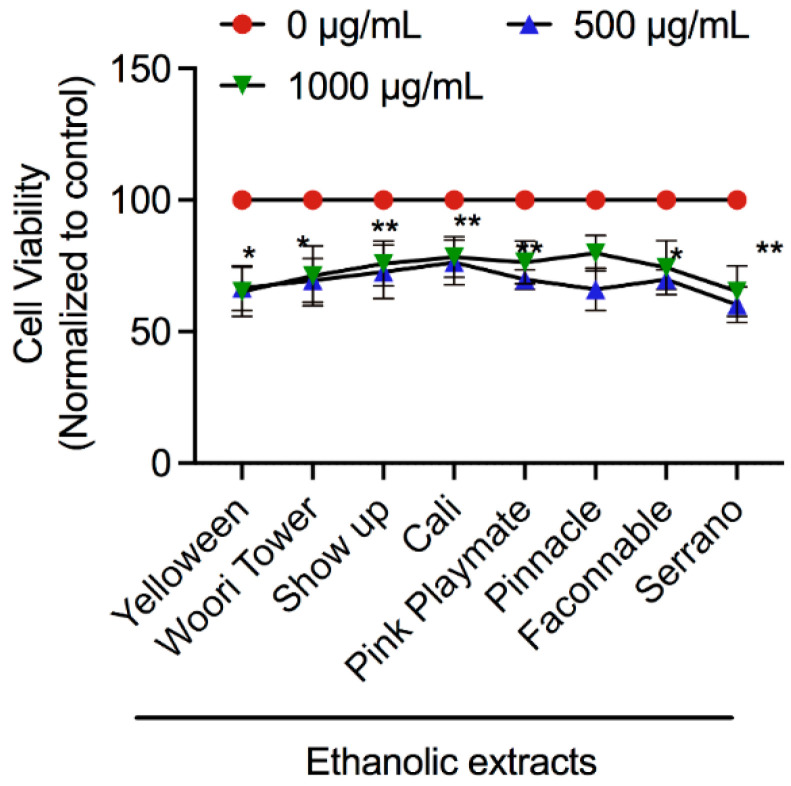
Effect of lily flower petal extracts on the LPS-stimulated RAW 264.7 cell metabolic viability. MTT assay in RAW 264.7 cells after treatment with various lily flower petal ethanolic extracts at 500 and 1000 µg/mL concentrations, as per indicated panel after 48 h. Significance has been considered * *p* < 0.05, ** *p* < 0.05.

To investigate the cytotoxicity of lily flower extracts in RAW 264.7 cells, cell metabolic viability was assessed using an MTT assay. The metabolic viability of the LPS-stimulated RAW 264.7 cells exposed to ethanolic extracts of different lily species is shown in Figure 3. Data indicate that cell viability was noticeably decreased after 48 h postincubation with extracts treatment. These findings indicate the possibility of anti-inflammatory effects in these extracts, as observed with the reduction in cellular growth of RAW 264.7 cells.

### 3.4. Effect of Lily Flower Petal Extracts on LPS-Induced NO Production

**Figure 4:**
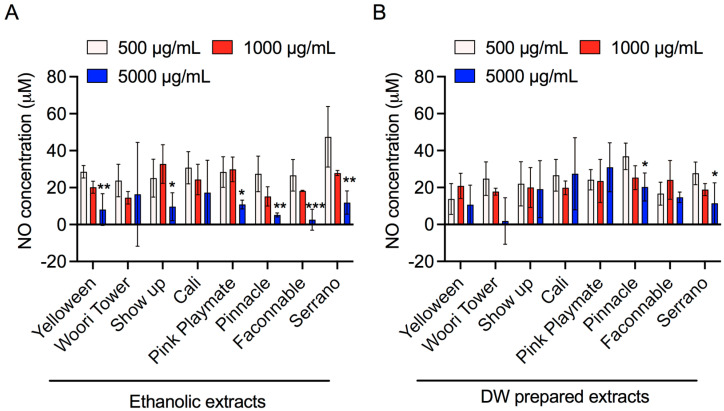
The effects of lily flower petal extracts on NO production in LPS-stimulated RAW264.7 cells. (**A**,**B**) These cells were exposed with or without extracts at various concentrations (500, 1000, 5000 µg/mL), and NO in the cell culture medium was analyzed using Griess reagent. Results are expressed as NO concentration (μM) as indicated in representative graphs. Significance has been considered * *p* < 0.05, ** *p* < 0.05, *** *p* < 0.05.

NO is a form of endogenous free radical species produced from l-arginine via nitric oxide synthase in eukaryotic cells. Generally, huge levels of NO are linked with various diseases and induced inflammation. Cells were pretreated with LPS (40 µg/mL) for 2 h and then treated with all extracts for additional incubation of 24 h. NO concentration was analyzed in the cell culture supernatants with the Griess reagent reaction. As shown in Figure 4, both ethanol and water extracts of Pinnacle and Serrano clearly showed significant decreases in NO production in a dose-dependent manner. Furthermore, the overall trend of experimental results showed that both ethanolic and DW extracts effectively decreased the NO production in a concentration-dependent fashion in LPS-stimulated RAW 264.7 cells (Figure 4). This result indicates that these lily flower extracts exert significant anti-inflammatory properties in those stimulated macrophage cells.

### 3.5. LC-MS-Characterization of Serrano Petal Extracts

**Figure 5:**
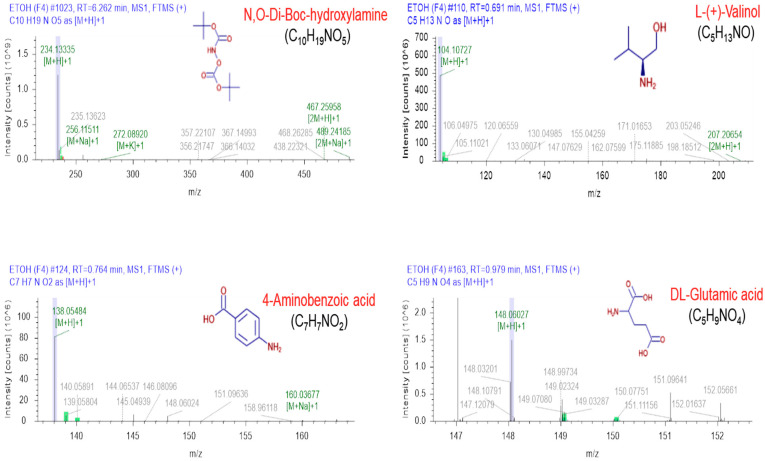
The mass spectra of major identified compounds present in the Serrano lilies petal extracts prepared in Ethanol.

**Figure 6:**
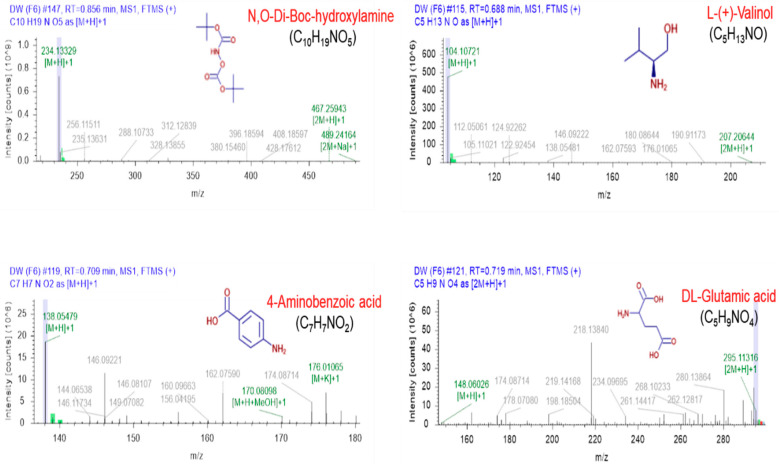
The mass spectra of major identified compounds present in the Serrano lilies petal extracts prepared in deionized water.

Generally, natural plants contain many phytochemicals. Therefore, it is important to identify the compounds present in the plant extracts before utilization. Since Serrano mainly showed similarity among water and ethanolic extracts, we investigated the compounds present mainly in those extracts, responsible for the above-observed activities. To this end, we performed the LC-MS analysis to explore the existing components of these extracts. The obtained LC-MS data show that four abundant compounds were detected in both extracts prepared in ethanol and DW, including N,O-Di-Boc-hydroxylamine, L-(+)-Valinol, 4-Aminobenzoic acid, and DL-Glutamic acid. The mass spectra of these compounds detected in both solvents are demonstrated in Figure 5 and Figure 6. Other information, such as the retention time, molecular weight, and molecular formula, are represented in Table 1.

## 4. Discussion

With the progress of people’s living standards, our aesthetic needs are increasing, which leads to the extensive usage of cosmetics. Furthermore, the use of cosmetic properties derived from natural resources is gradually being encouraged because of their suitability for sustainable production and longlisting health benefits [1]. It has been reported that Liliaceae is one of the foremost plant families for natural resources-based cosmetic formulations [36]. Additionally, the high demand for oral supplements to attain the best results from the inside out offers an opportunity for medicinal lilies in cosmetic preparations [37]. Currently, in the cosmetic industry, lilies are typically added as extracts, few in powder form, and most of them are obtained from wild species. Consequently, it is crucial to investigate new *Lilium* materials for their function in the cosmetic field. Bulbus lily has been well-recognized to have natural antioxidants, for example, polyphenols, flavonoids, ascorbic acid, including anthocyanins and ethanol soluble carotenoids, and chlorophyll [38]. Moreover, Pérez-Gálvez et al. recently suggested that carotenoids and chlorophyll have strong antioxidant activity [39]. In our study, the antioxidant capacity in eight representative lilies, including different hybrid lilies, was studied together for the first time. Specifically, Yelloween, Pink playmate, Pinnacle, and Faconnable petal ethanol extracts clearly showed substantial antioxidant activities in a dose-dependent manner. In contrast, pink playmate and Pinnacle petal water extracts did not demonstrate antioxidant activities, whereas Yelloween and Faconnable petal water extracts showed significant antioxidant activities.

Numerous physical, chemical, and biological agents can produce inflammation with an elevated risk of diseases [40]. It is well-known that inflammation is a critical physiological defense phenomenon caused by infection or exposure to endotoxins, for example, LPS [41]. LPS is produced by bacteria (Gram-negative) and is an active inducer of inflammatory immune responses in eukaryotic cells [42]. At the time of the inflammation process, high NO generation is a prime target in drug development for inflammatory diseases [43]. NO causes harmful effects with tissue injury, apoptosis, and sepsis [44]. Consequently, several studies are currently ongoing to develop inhibitors using medicinal plant products to cure or counteract chronic inflammatory disorders with minimal toxicity [45]. In this study, levels of NO were determined following exposure to different concentrations of lily flower petal extracts. Ethanolic and DW extracts showed a dose-dependent suppression of LPS-stimulated NO production; however, a higher effect was observed at the maximum treated concentration (5000 μg/mL) (Figure 4). These results suggest that ethanol-soluble components would play a role in the powerful antioxidant and anti-inflammatory effects of lily flower petal extracts. In agreement, interestingly, our data disclosed that the lily flower petal extracts could reduce 40–50% viability of LPS-stimulated RAW264.7 cells, suggesting that these extracts can exhibit functional activity (Figure 3).

Apart from the antioxidant and anti-inflammatory activities, the inhibition of tyrosinase activity has a vital role in preventing melanin accumulation in the skin [32]. Hence, tyrosinase inhibitors are an appealing target in cosmetics formulations as well as skin pigmentation disorders treatments. We observed that lily bulb flower extracts have good potential for inhibiting tyrosine kinase activity, mainly in ethanolic and water extracts (Figure 2). In summary, we revealed that lily flower petal extracts have high antioxidant enzyme properties, which prevent NO generation in inflamed RAW 264.7 macrophage cells. Keeping with these purposes, this study planned to determine the responsible phytocompounds in potential lily petal extracts through LC-MS analysis for the observed above effects. Chromatography procedures perform a key role in phytochemicals profiling and are consistently employed for the detailed analysis of biologically and pharmaceutically active ingredients present in plant extracts. Our results confirmed the existence of several phytochemicals (~1000), including phenols, flavonoids, saponins, etc., in Serrano lily petal extracts prepared in ethanol and water, suggesting the pharmaceutical significance of these extracts. Among them, four phytochemicals named N,O-Di-Boc-hydroxylamine, L-(+)-Valinol, 4-Aminobenzoic acid, and DL-Glutamic acid are most prominent in Serrano lily petal extracts prepared in both solvents. It is well-accepted that the biological consequences of plant extracts basically depend upon the selection of extraction procedures. The choice of solvent used has major significance and must be standardized prior to final use. Based on our studies, we can conclude that both ethanol and water extracts work better in the case of Serrano lily petals; however, more detailed experiments are required.

## 5. Conclusions

Thus, these lily extracts could be a potent anti-inflammatory agent with a whitening effect and antioxidant activities. Moreover, several reports have demonstrated that, apart from the bulbs, other parts of lilies are also enriched in polyphenols and other phytochemicals. Therefore, it is quite promising to use such parts to extract supplements for cosmetics preparations. Overall, we conclude that lily petal extracts are favorable candidates for further progress as antioxidant or pharmaceutical supplements. Further studies should be focused on the evaluations of economic benefit, and in vivo testing of such lily petal extracts should be conducted prior to their commercial utilization.

## Figures and Tables

**Table 1:** Characteristics of detected compounds from Serrano lily’s petal extracts by LC-MS.

No.	Compound	RT (Ethanol)/min	RT (DW)/min	Molecular Formula	Average Mass (g/mol)	Molecular Formula
1	N,O-Di-Boc-hydroxylamine	6.26	0.86	C_10_H_19_NO_5_	233.13	C_10_H_19_NO_5_
2	L-(+)-Valinol	0.69	0.69	C_5_H_13_NO	103.10	C_5_H_13_NO
3	4-Aminobenzoic acid	0.77	0.71	C_7_H_7_NO_2_	137.05	C_7_H_7_NO_2_
4	DL-Glutamic acid	0.98	0.72	C_5_H_9_NO_4_	147.05	C_5_H_9_NO_4_

## Data Availability

All the data related to the manuscript has been included here.
